# Three New Sesquiterpenoids from the Algal-Derived Fungus *Penicillium chermesinum* EN-480

**DOI:** 10.3390/md18040194

**Published:** 2020-04-07

**Authors:** Xue-Yi Hu, Xiao-Ming Li, Sui-Qun Yang, Hui Liu, Ling-Hong Meng, Bin-Gui Wang

**Affiliations:** 1 Key Laboratory of Experimental Marine Biology, Institute of Oceanology, Chinese Academy of Sciences, Nanhai Road 7, Qingdao 266071, China; huxueyi14@mails.ucas.ac.cn (X.-Y.H.); lixmqdio@126.com (X.-M.L.); suiqunyang@163.com (S.-Q.Y.); liuhui1625@163.com (H.L.); 2 Laboratory of Marine Biology and Biotechnology, Qingdao National Laboratory for Marine Science and Technology, Wenhai Road 1, Qingdao 266237, China; 3 College of Earth Science, University of Chinese Academy of Sciences, Yuquan Road 19A, Beijing 100049, China; 4 Center for Ocean Mega-Science, Chinese Academy of Sciences, Nanhai Road 7, Qingdao 266071, China

**Keywords:** *Penicillium chermesinum*, algal-derived fungus, sesquiterpenoid, antimicrobial activity

## Abstract

Secondary metabolites obtained from marine-derived fungi are rich sources of drug candidates. Three new sesquiterpenoids, chermesiterpenoids A–C (**1**–**3**), along with four known alkaloids (**4**–**7**), were isolated and identified from the marine red algal-derived fungus *Penicillium chermesinum* EN-480. The structures of these new sesquiterpenoids were elucidated using detailed analysis of the NMR data and their relative configurations were elucidated using nuclear overhauser effect spectroscopy (NOESY) spectra as well as gauge-independent atomic orbital (GIAO) NMR shift calculations and DP4+ probability analysis. Their absolute configurations were determined using electronic circular dichroism (ECD) calculations and modified Mosher’s method. Compounds **2** and **3** exhibited potent activities against human and aquatic pathogenic bacteria and plant pathogenic fungi.

## 1. Introduction

**Figure 1:**
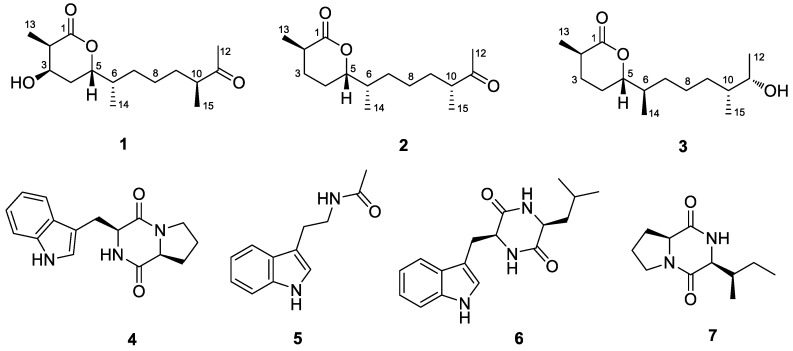
Structures of compounds **1**–**7**.

Marine-derived fungi are rich sources of diverse and bioactive secondary metabolites [1,2]. Among them, the species of the genus *Penicillium* play an important role and have increasingly attracted attention [3]. Our current research focuses on *Penicillium chermesinum* EN-480, a fungal strain isolated from the fresh tissue of the marine red alga *Pterocladiella tenuis* [4]. To date, meroterpenoids [4], cytotoxic metabolites [5], azaphilones [6], terphenyls [6], and plastatin [7] have been obtained from the species *P. chermesinum*. Some of them exhibited antibacterial [4] and enzyme inhibitory [6] activities. In our previous research, we obtained antimicrobial meroterpenoids from *P. chermesinum* EN-480 [4], and we recently tried to change the fermentation process to obtain more antibacterial secondary metabolites. As a result, we isolated three new sesquiterpenoids (**1**–**3**) as well as four known alkaloids, brevianmide F (**4**) [8], *N*-acetyltryptamine (**5**) [9], cyclo Trp-Ile (**6**) [10], and cyclo Ile-Pro (**7**) [11] (Figure 1). Compounds **1**–**3** were formed by esterification and cyclizing of catenulate farnesyl probably, and this is the first time this type of sesquiterpenoid from natural products has been described. The absolute configurations of this kind of secondary metabolites were difficult to determine because the alkyl chains were flexible and there were no conjugated chromophores in the compounds. Thus, we combined DP4+ probability analysis [12,13], electronic circular dichroism (ECD) calculations, and modified Mosher’s method [14,15] to solve this problem. Herein, details of the isolation, structure determination, and biological activities of these compounds are described.

## 2. Results and Discussion

### 2.1. Structure Elucidation of the New Compounds

**Figure 2:**
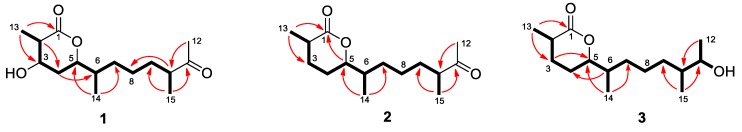
Key COSY (homonuclear chemical shift correlation spectroscopy) (bold line) and HMBC (heteronuclear multiple bond correlation) (arrow) correlations of compounds **1**–**3**.

Compound **1** was obtained as a yellowish solid. Its molecular formula was determined as C_15_H_26_O_4_ based on the (+)-HRESIMS (high resolution electrospray ionization mass spectroscopy) data (Figure S1, Supplementary Material), which has three degrees of unsaturation (index of hydrogen deficiency) [16]. The ^13^C NMR and DEPT (distortionless enhancement by polarization transfer) data (Table 1 and Figure S3) presented 15 carbon resonances, including four methyls, four methylenes, five methines (including two oxygenated), and two quaternary carbons (with one ketone carbonyl and one ester carbonyl). The ^1^H NMR data (Table 1 and Figure S2) revealed the presence of four methyl signals at *δ*_H_ 2.09 (H-12, 3H, s), 1.10 (H-13, 3H, d, *J* = 7.1 Hz), 0.84 (H-14, 3H, d, *J* = 6.9 Hz), and 0.98 (H-15, 3H, d, *J* = 6.8 Hz), as well as five methines (including two oxygenated at *δ*_H_ 4.44 (H-5, 1H, m) and 3.95 (H-3, 1H, m)) in compound **1**. The ^1^H and ^13^C NMR data indicated that it is a sesquiterpenoid and the planar structure was further determined and fully supported using the COSY (homonuclear chemical shift correlation spectroscopy) and HMBC (heteronuclear multiple bond correlation) correlations (Figure 2, Figure S4 and S5). Compound **1** was possibly formed by the oxidation and esterification of farnesyl. This is the first time this type of sesquiterpenoid, which contains a pentanolactone moiety, has been described.

**Figure 3:**
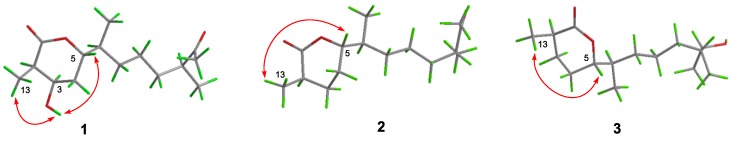
NOESY (nuclear overhauser effect spectroscopy) correlations of compounds **1**–**3**.

**Figure 4:**
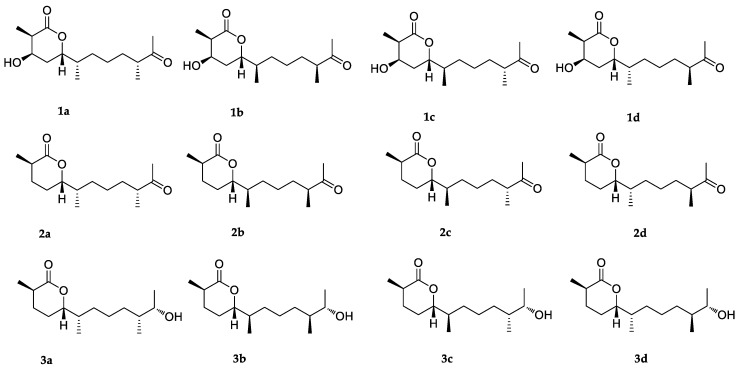
Structures of possible isomers for DP4+ probability analysis of compounds **1**–**3**.

Based on the NOESY (nuclear overhauser effect spectroscopy) spectrum (Figure S6), only a part of the relative configuration in compound **1** could be determined. The NOESY correlations from 3-OH to H_3_-13 and H-5 confirmed that they were on the same side of **1** (Figure 3). However, NOESY data cannot be used to determine the relative configuration of the methyl groups at the alkyl chain due to the flexibility of this moiety. Thus, DP4+ probability analysis was used to indicate the relative configuration at C-6 and C-10. This method was used to assign the relative configurations of organic molecules employing GIAO (gauge-independent atomic orbital) NMR shift calculations [17,18]. Based on a DP4+ protocol [19], both proton and carbon data of four possible isomers were calculated, and the results were analyzed with the experimental values. The statistical comparison showed that the isomer **1d** was the equivalent structure with a probability of 99.90% (Figure 4 and Figure S17). Thus, the results indicated that the relative configuration of **1** is 2R*, 3R*, 5S*, 6S*, and 10S*. 

**Figure 5:**
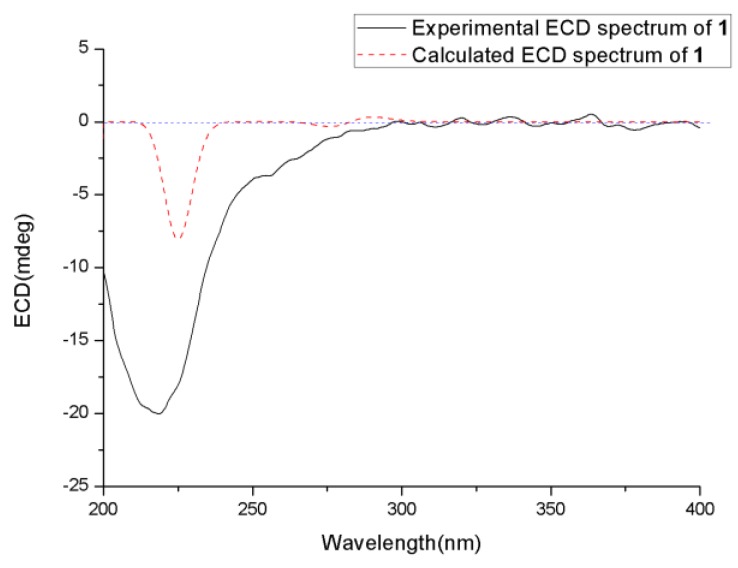
Experimental and calculated ECD (electronic circular dichroism) spectra of **1**.

The absolute configuration of **1** was determined using ECD calculation. Gaussian 09.10 was used to perform conformational search and geometry optimization. The obtained minimum energy conformers were calculated using the time-dependent density functional theory (TDDFT) method at the B3LYP/6-31G (d) PCM (polarizable continuum model) at MeOH level for their ECD spectra. The experimental ECD spectrum of **1** matched well with that calculated for 2*R*, 3*R*, 5*S*, 6*S*, and 10*S* configurations, which showed negative cotton effect (CE) at approximate 218 nm (Figure 5). Thus, the structure of **1** was determined and was named chermesiterpenoid A.

Compound **2** was obtained as a yellowish solid. Its molecular formula was determined as C_15_H_26_O_3_, with one O atom less than that of **1**, on the basis of (+)-HRESIMS data. The ^1^H and ^13^C NMR spectra of **2** are similar to compound **1**, indicating that it is also a sesquiterpenoid. However, the NMR data revealed that the oxygenated methine signal at C-3 (*δ*_C_ 65.8; *δ*_H_ 3.95) of compound **1** was replaced by a methylene signal (*δ*_C_ 27.9; *δ*_H_ 1.90/1.56) in **2** (Table 1 and Figure S7 and S8), showing that the hydroxyl group at C-3 of **1** disappeared in that of **2**. The observed COSY correlations (Figure S9) from H-3 to H-2 and H-4, as well as the HMBC correlation (Figure S10) from H_3_-13 to C-3, provided further evidence (Figure 2).

**Figure 6:**
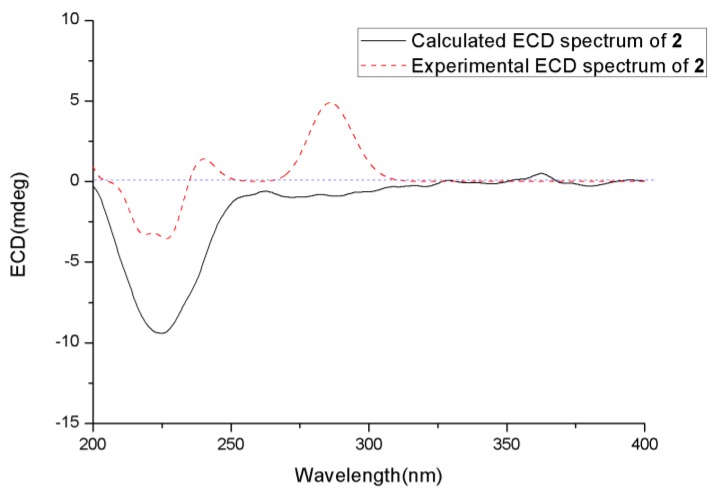
Experimental and calculated ECD spectra of **2**.

The relative configuration of **2** was also determined using NOESY spectrum (Figure S11) and DP4+ probability analysis. The NOESY correlation between H_3_-13 and H-5 indicated that they were on the same side. The DP4+ probability analysis of both proton and carbon data showed that the isomer **2a** was the equivalent structure with the probability of 100.00% (Figure 4 and Figure S18). The absolute configuration of **2** was also determined using the TDDFT-ECD calculation, and the experimental ECD spectrum of **2** showed excellent agreement with that calculated for 2*R*, 5*S*, 6*S*, and 10*R* configurations (Figure 6). The structure of **2** was thus determined and was named chermesiterpenoid B.

Compound **3** was also obtained as a yellowish solid. Its molecular formula was determined as C_15_H_28_O_3_, with two H atoms more than that of **2** and with two degrees of unsaturation (index of hydrogen deficiency) [16], on the basis of (+)-HRESIMS data. The NMR data of **3** (Figures S12 and S13) were very similar to those of compound **2** except that the ketone carbonyl at C-11 (*δ*_C_ 211.8) in **2** was missing and a hydroxyl methine (*δ*_C_ 68.8; *δ*_H_ 3.48, CH-11) in **3** appeared, indicating that the ketone carbonyl at C-11 was replaced by the hydroxyl group. The chemical shift of H_3_-12 was shielded from *δ*_H_ 2.09 (3H, s) in **2** to *δ*_H_ 0.97 (3H, d, *J* = 6.3 Hz) in **3** in the ^1^H NMR spectrum. The planar structure was further confirmed using COSY and HMBC correlations (Figure 2, Figures S14 and S15).

**Figure 7:**
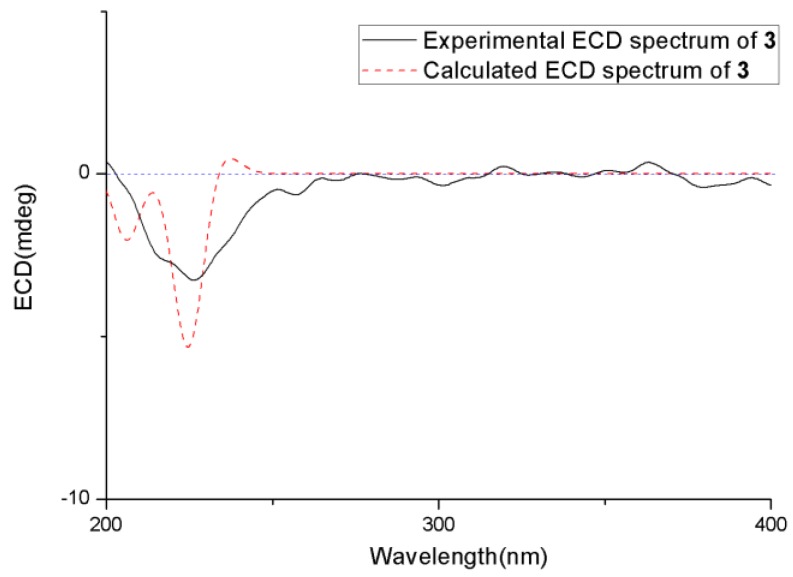
Experimental and calculated ECD spectra of **3**.

**Figure 8:**
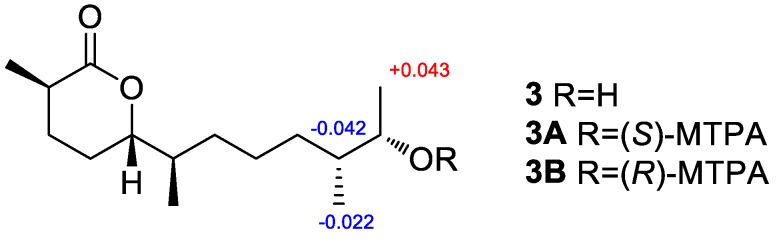
Δ*δ* values (Δ*δ* (in ppm) = *δ_S_ – δ_R_*) obtained for the (*S*)-and (*R*)-MTPA (*α*-methoxy-*α*-(trifluoromethyl) phenylacetyl) esters of compound **3**.

The relative and absolute configurations of **3** were determined using NOESY data, ECD calculation, the modified Mosher’s method, and DP4+ probability analysis. First, the NOESY correlation (Figure S16) from H_3_-13 to H-5 indicated that they were on the same side. Then, the absolute configuration of C-2 and C-5 was determined using the ECD calculation. The experimental ECD spectrum of **3** agreed well with that calculated for 2*R* and 5*S* configurations (Figure 7). After that, modified Mosher’s method was used to determine the absolute configuration of C-11, and the observed Δ*δ* (*δ_S_-δ_R_*) values of *α*-methoxy-*α*-(trifluoromethyl) phenylacetyl (MTPA) esters suggested that the absolute configuration at C-11 was *S* (Figure 8). Finally, both proton and carbon data of four possible isomers with different absolute configuration at C-6 and C-10 were calculated using the DP4+ probability analysis, and the result showed that the isomer **3c** was the equivalent structure with a probability of 100.00% (Figure 4 and Figure S19). The absolute configuration of **3** was determined as 2*R*, 5*S*, 6*R*, 10*R*, and 11*S*. Thus, the structure of **3** was determined and was named chermesiterpenoid C.

### 2.2. Biological Activities of the Isolated Compounds

The isolated new compounds (**1**–**3**) were examined for antimicrobial activities against several human-, aqua-, and plant-pathogenic microbes. Compound **2** showed activities against the aquatic pathogens *Vibrio anguillarum*, *Vibrio parahaemolyticus*, *Micrococcus luteus*, and human pathogen *Escherichia* *coli* with minimum inhibitory concentration (MIC) values of 0.5, 16, 64, and 64 μg/mL, respectively, which are comparable to that of the positive control, chloromycetinum (MIC = 0.5, 1, 0.5, and 1 μg/mL, respectively). Similarly, compound **3** showed activities against the aquatic pathogens *V. anguillarum*, *V. parahaemolyticus*, and *M. luteus* with MIC values of 1, 32, and 64 μg/mL, respectively. Compounds **1**–**3** exhibited activity against the plant pathogenic fungus *Colletottichum gloeosporioides* with MIC values of 64, 32, and 16 μg/mL, respectively, whereas the positive control amphotericin B had a MIC value of 1.0 μg/mL. 

These data indicated that compound **1**, with a hydroxyl group at C-3, has weaker antimicrobial activities than compounds **2** and **3**, regardless of whether against aquatic and human pathogens or plant pathogenic fungi. Compound **2** has somewhat stronger activities against aquatic and human pathogens but weaker activity against plant pathogenic fungus *C. gloeosporioides* than compound **3**, probably due to the different degree of oxidation at C-11.

## 3. Materials and Methods 

### 3.1. General

UV spectra were obtained using a PuXi TU-1810 UV-visible spectrophotometer (Shanghai Lengguang Technology Co. Ltd., Shanghai, China). Optical rotations were recorded using an Optical Activity AA-55 polarimeter (Optical Activity Ltd., Cambridgeshire, U.K.). ECD spectra were evaluated on a JASCO J-715 spectropolarimeter (JASCO, Tokyo, Japan). The 1D and 2D NMR spectra were determined using a Bruker Avance 500 spectrometer (Bruker Biospin Group, Karlsruhe, Germany). A VG Autospec 3000 (VG Instruments, London, U.K.) was used to measure mass spectra. Analytical and semi-preparative HPLC were performed on a Dionex HPLC system equipped with a P680 pump (Dionex, Sunnyvale, CA, USA), ASI-100 automated sample injector (Dionex, Sunnyvale, CA, USA), and UVD340U multiple wavelength detector (Dionex, Sunnyvale, CA, USA) controlled by Chromeleon software (version 6.80) (Dionex, Sunnyvale, CA, USA). Column chromatography (CC) was performed with silica gel (100–200 and 200–300 mesh; Qingdao Haiyang Chemical Factory, Qingdao, China), Sephadex LH-20 (18–110 μm, Merck, Darmstadt, Germany), and Lobar LiChroprep RP-18 (40–60 μm, Merck, Darmstadt, Germany).

### 3.2. Fungal Material

The fungus *Penicillium chermesinum* EN-480 was isolated from the fresh tissue of marine red algal *Pterocladiella tenuis*, collected from Rongcheng, Shandong province, in China in July 2014. The fungus was identified as *Penicillium chermesinum* using sequence analysis of the ITS (internal transcribed spacer) region of its 18S rDNA as described previously [20]. The resulting sequence data obtained were deposited in GenBank (accession no. KT119566). The strain is preserved at the key Laboratory of Experimental Marine Biology, Institute of Oceanology, Chinese Academy of Sciences.

### 3.3. Fermentation

The fermentation was conducted dynamically in a 500 L fermentation tank (containing 1% glucose, 2% mannose, 2% maltose, 0.3% yeast extract powder, 0.1% corn flour, 1% monosodium glutamate, 0.05% K_2_HPO_4_, 0.03% MgSO_4_**•**7H_2_O, and 300 L naturally sourced and filtered seawater, pH 6.5−7.0) for six days at 28 °C. 

### 3.4. Extraction and Isolation 

After incubation, the fermentation broth was exhaustively extracted with EtOAc (ethyl acetate) three times. The combined EtOAc solution was concentrated under reduced pressure to yield an extract (155.3 g). 

The organic extract was subjected to vacuum liquid chromatography (VLC) eluting with different solvents of increasing polarity from petroleum ether (PE) to MeOH to yield 9 fractions (Fr. 1–9). Fr. 6 (20 g), eluted with PE-EtOAc (1:1), was purified using reverse-phase column chromatography (CC) over a Lobar LiChroprep RP-18 (40–60 μm, Merck, Darmstadt, Germany) with a MeOH-H_2_O gradient (from 10:90 to 100:0) to yield four subfractions (Fr. 6-1–6-4). Fr. 6-1, eluted with MeOH-H_2_O (20:80), was further purified using CC on silica gel (eluted with CH_2_Cl_2_-MeOH, 100:1) and Sephadex LH-20 (18–110 μm, Merck, Darmstadt, Germany) (MeOH) to obtain compound **1** (17.4 mg). Fr. 6-2 (eluted with MeOH-H_2_O (40:60)) was further purified using prep. TLC (plate: 20 × 20 cm, developing solvents: CH_2_Cl_2_-MeOH, 50:1) and Sephadex LH-20 (18–110 μm, Merck, Darmstadt, Germany) (MeOH) to produce compounds **4** (5.0 mg), **5** (4.5 mg), and **6** (3.0 mg). Fr. 6-3 (eluted with MeOH-H_2_O (50:50)) was further purified using CC on silica gel (eluted with CH_2_Cl_2_-MeOH, 150:1) and prep. TLC (plate: 20 × 20 cm, developing solvents: CH_2_Cl_2_-MeOH, 100:1) to produce compound **2** (19.1 mg). Fr. 6-4 (eluted with MeOH-H_2_O (60:40)) was further purified using CC on silica gel (eluted with CH_2_Cl_2_-MeOH, 200:1 to 100:1) and Sephadex LH-20 (18–110 μm, Merck, Darmstadt, Germany) (MeOH) to produce compounds **3** (10.0 mg) and **7** (7.2 mg).

Chermesiterpenoid A (**1**): yellowish solid; ${\left[\alpha \right]}_{D}^{20}$ +6.25 (*c* 0.16, MeOH); ECD λ_max_ (Δε) 218.5 (−1.64), ^1^H and ^13^C NMR data, see Table 1; HRESIMS *m/z* 271.1906 [M + H]^+^, 288.2172 [M + NH_4_]^+^ (calcd for C_15_H_26_O_4_, 270.1831).

Chermesiterpenoid B (**2**): yellowish solid; ${\left[\alpha \right]}_{D}^{20}$ +10.00 (*c* 0.10, MeOH); ECD λ_max_ (Δε) 224.5 (−0.72) nm;^1^H and ^13^C NMR data, see Table 1; HRESIMS *m/z* 255.1951 [M + H]^+^, 277.1765 [M + Na]^+^ (calcd for C_15_H_26_O_3_, 254.1882).

Chermesiterpenoid C (**3**): yellowish solid; ${\left[\alpha \right]}_{D}^{20}$ +3.70 (*c* 0.27, MeOH); ECD λ_max_ (Δε) 226.5 (−0.25) nm; ^1^H and ^13^C NMR data, see Table 1; HRESIMS *m/z* 257.2113 [M + H]^+^, 279.1930 [M + Na]^+^ (calcd for C_15_H_28_O_3_, 256.2038). 

### 3.5. Computational NMR Chemical Shift Calculation and DP4+ Analyses

All theoretical calculations were performed via the Gaussian 09 program package. Conformational searches for all possible isomers were conducted via molecular mechanics using the Merck molecular force field (MMFF) method with Macromodel software (Schrödinger, LLC, New York City, USA) and the corresponding stable conformer, from which distributions higher than 2% were collected. After this, B3LYP/6-31G (d) PCM level in DMSO (dimethyl sulfoxide) was used to optimize the conformers. Then, the NMR shielding tensors of all optimized conformers were calculated using the DFT method at mPW1PW91\6-31+G (d) PCM level on DMSO and then averaged based on Boltzmann distribution theory [19]. NMR chemical shifts were calculated using an equation described previously [21]. Finally, the NMR chemical shifts and shielding tensors (^1^H and ^13^C) were analyzed and compared with the experimental chemical shifts using DP4+ probability [19].

### 3.6. ECD Calculation

Conformational searches were conducted via molecular mechanics using the MMFF method in Macromodel software (Schrödinger, LLC, New York City, NY, USA), and the geometries were reoptimized at B3LYP/6-31G(d) PCM/MeOH level using Gaussian 09 software [22] to generate the energy-minimized conformers. After, the optimized conformers were used to calculate ECD spectra using TDDFT at CAM-B3LYP and BH&HLYP/TZVP; the solvent effects of the MeOH solution were estimated at the same DFT level using the SCRF/PCM method.

### 3.7. Modified Mosher‘s Method 

Compound **3** (2.0 mg) was separated equally into two flasks, and both were dried completely. Anhydrous pyridine (400 μL), dimethylaminopyriridine (DMAP, 4 mg), and (*S*)-(+)-α-methoxy-α-(trifluoromethyl) phenylacetyl chloride (10 μL) or (*R*)-(−)-α-methoxy-α-(trifluoromethyl) phenylacetyl chloride (10 μL) were added to the two flasks. Then, the flasks were shaken carefully to mix the samples, and the reaction was performed at room temperature for 12 hours. After that, three drops of water were added to stop the reaction. The reaction mixtures were reduced pressure distillated and purified using prep. TLC (plate: 20 × 20 cm, developing solvents: CH_2_Cl_2_-MeOH, 50:1) to produce (*R*)- and (*S*)-MTPA ester derivatives, respectively, and the ^1^H NMR spectra and COSY were detected [23].

### 3.8. Antimicrobial Assay

Antimicrobial assays against human- and aqua-pathogenic microbes *Aeromonas hydrophilia*, *Edwardsiella megi*, *Edwardsiella tarda*, *Escherichia coli*, *Micrococcus luteus*, *Pseudomonas aeruginosa*, *Vibrio alginolyticus*, *V. anguillarum*, *V. harveyi*, *V. parahaemolyticus*, *V. vulnificus*, and plant pathogenic fungi *Bipolaris sorokiniana*, *Colletottichum gloeosprioides*, *Fusarium graminearum*, *F. oxysporum*, *Phytophthora nicotiana*, *Physalospora piricola*, and *Valsa mali* were conducted using the well diffusion method [24]. Chloromycetin was used as a positive control for the bacteria, whereas amphotericin B was used as a positive control for the fungi.

## 4. Conclusions

Three new types of sesquiterpenoids (**1**–**3**) and four known alkaloids (**4**–**7**) were isolated from the marine red algal-derived fungus *Penicillium chermesinum* EN-480. The structures of these new sesquiterpenoids were elucidated using detailed interpretation of the spectroscopic data. The absolute configurations were elucidated using DP4+ probability analysis, ECD calculations, and modified Mosher’s method. Compounds **2** and **3** exhibited prominent activity against aquatic pathogenic bacteria *Vibrio anguillarum*, *V. parahaemolyticus*, and *Micrococcus luteus*, human pathogen *Escherichia* *coli*, and plant pathogenic fungus *Colletottichum gloeosporioides*.

## Figures and Tables

**Table 1:** ^1^H (125 MHz) and ^13^C NMR (500 MHz) data of compounds **1**–**3** (recorded in DMSO (dimethyl sulfoxide)-*d*_6_, *δ* in ppm).

Pos.	1		2		3	
	^1^H (*J* in Hz)	^13^C	^1^H (*J* in Hz)	^13^C	^1^H (*J* in Hz)	^13^C
1		173.5, C		173.6, C		173.6, C
2	2.52, overlap	41.0, CH	2.40, dt (13.2, 6.7)	35.5, CH	2.40, m	35.5, CH
3	3.95, m	65.8, CH	*α* 1.90, m, *β* 1.56, overlap	27.9, CH_2_	*α* 1.90, ddd (8.5, 6.2, 2.9) *β* 1.53, m	27.7, CH_2_
4	*α* 1.73, m, *β* 1.82, m	23.9, CH_2_	*α* 1.78, d (10.7) *β* 1.57, overlap	27.6, CH_2_	*α* 1.78, m *β* 1.51, m	24.5, CH_2_
5	4.44, m	79.2, CH	4.16, m	84.4, CH	4.17, m	84.5, CH
6	1.68, m,	36.7, CH	1.64, m	37.0, CH	1.64, m	37.2, CH
7	1.14, m	31.3, CH_2_	1.08, m	31.3, CH_2_	1.05, m	31.8, CH_2_
8	1.26, m	32.0, CH_2_	1.27, m	24.6, CH_2_	1.40, m	24.3, CH_2_
9	α 1.14, m, *β* 1.55, m	32.4, CH_2_	*α* 1.23, m, *β* 1.55, overlap	32.4, CH_2_	*α* 1.03, m *β* 0.96, m	32.4, CH_2_
10	2.52, overlap	46.0, CH	2.50, overlap	46.0, CH	1.31, m	40.0, CH
11		211.8, C		211.8, C	3.48, dt (10.7, 5.4)	68.8, CH
12	2.09, s	27.9, CH_3_	2.09, s	23.9, CH_3_	0.97, d (6.3)	20.3, CH_3_
13	1.10, d (7.1)	12.8, CH_3_	1.14, d (7.0)	17.0, CH_3_	1.13, d (7.0)	17.1, CH_3_
14	0.84, d (6.9)	14.4, CH_3_	0.84, d (6.8)	14.4, CH_3_	0.85, d (6.8)	14.5, CH_3_
15	0.98, d (6.8)	15.9, CH_3_	0.99, d (6.9)	15.9, CH_3_	0.79, d (6.7)	14.5, CH_3_
3-OH	5.20, br s					
11-OH					4.21, d (4.7)	

## Supplementary Materials

The following are available online at https://www.mdpi.com/1660-3397/18/4/194/s1, Figure S1: HRESI mass spectrum of compound **1**. Figure S2: ^1^H NMR (500 MHz, DMSO-*d*_6_) spectrum of compound **1**. Figure S3: ^13^C NMR (125 MHz, DMSO-*d*_6_) and DEPT spectra of compound **1**. Figure S4: COSY spectrum of compound **1**. Figure S5: HMBC spectrum of compound **1**. Figure S6: NOESY spectrum of compound **1**. Figure S7: ^1^H NMR (500 MHz, DMSO-*d*_6_) spectrum of compound **2**. Figure S8: ^13^C NMR (125 MHz, DMSO-*d*_6_) and DEPT spectra of compound **2**. Figure S9: COSY spectrum of compound **2**. Figure S10: HMBC spectrum of compound **2**. Figure S11: NOESY spectrum of compound **2**. Figure S12: ^1^H NMR (500 MHz, DMSO-*d*_6_) spectrum of compound **3**. Figure S13: ^13^C NMR (125 MHz, DMSO-*d*_6_) and DEPT spectra of compound **3**. Figure S14: COSY spectrum of compound **3**. Figure S15: HMBC spectrum of compound **3**. Figure S16: NOESY spectrum of compound **3**. Figure S17: DP4+ probability Excel sheets of compound **1**. Figure S18: DP4+ probability Excel sheets of compound **2**. Figure S19: DP4+ probability Excel sheets of compound **3**.
