# Supplementary material for: Three New Sesquiterpenoids from the Algal-Derived Fungus Penicillium chermesinum EN-480
Source: Mar Drugs. 2020 Apr 7;18(4):194. doi: 10.3390/md18040194 (PMC7230704; doi:10.3390/md18040194)
Supplement: Supplementary file 1 [file marinedrugs-18-00194-s001.pdf]

Supplementary Material

Three New Sesquiterpenoids from the Algal-Derived Fungus *Penicillium chermesinum* EN-480

Xue-Yi Hu [1,2,3], Xiao-Ming Li [1,2], Sui-Qun Yang [1,2], Hui Liu [1], Ling-Hong Meng [1,2,*] and Bin-Gui Wang [1,2,4,*]

[1] Key Laboratory of Experimental Marine Biology, Institute of Oceanology, Chinese Academy of Sciences, Nanhai Road 7, Qingdao 266071, China; E-Mails: huxueyi14@mails.ucas.ac.cn (X.-Y.H.); lixmqdio@126.com (X.-M.L.); suiqunyang@163.com (S.-Q.Y.); liuhui1625@163.com (H.L.)
[2] Laboratory of Marine Biology and Biotechnology, Qingdao National Laboratory for Marine Science and Technology, Wenhai Road 1, Qingdao 266237, China
[3] College of Earth Science, University of Chinese Academy of Sciences, Yuquan Road 19A, Beijing 100049, China
[4] Center for Ocean Mega-Science, Chinese Academy of Sciences, Nanhai Road 7, Qingdao 266071, China
* Correspondence: menglh@ms.qdio.ac.cn (L.-H.M.); Tel: +86-532-8289-8890 (L.-H.M.); wangbg@ms.qdio.ac.cn (B.-G.W.); Tel.: +86-532-8289 8553 (B.-G.W.)

Content

Figure S1. HRESI mass spectrum of compound **1**.

Figure S2. ^{1}H NMR (500 MHz, DMSO-d_6) spectrum of compound **1**.

Figure S3. ^{13}C NMR (125 MHz, DMSO-d_6) and DEPT spectra of compound **1**.

Figure S4. COSY spectrum of compound **1**.

Figure S5. HMBC spectrum of compound **1**.

Figure S6. NOESY spectrum of compound **1**.

Figure S7. ^{1}H NMR (500 MHz, DMSO-d_6) spectrum of compound **2**.

Figure S8. ^{13}C NMR (125 MHz, DMSO-d_6) and DEPT spectra of compound **2**.

Figure S9. COSY spectrum of compound **2**.

Figure S10. HMBC spectrum of compound **2**.

Figure S11. NOESY spectrum of compound **2**.

Figure S12. ^{1}H NMR (500 MHz, DMSO-d_6) spectrum of compound **3**.

Figure S13. ^{13}C NMR (125 MHz, DMSO-d_6) and DEPT spectra of compound **3**.

Figure S14. COSY spectrum of compound **3**.

Figure S15. HMBC spectrum of compound **3**.

Figure S16. NOESY spectrum of compound **3**.

Figure S17. DP4+ probability Excel sheets of compound **1**.

Figure S18. DP4+ probability Excel sheets of compound **2**.

Figure S19. DP4+ probability Excel sheets of compound **3**.

Figure S1. HRESI mass spectrum of compound **1.**

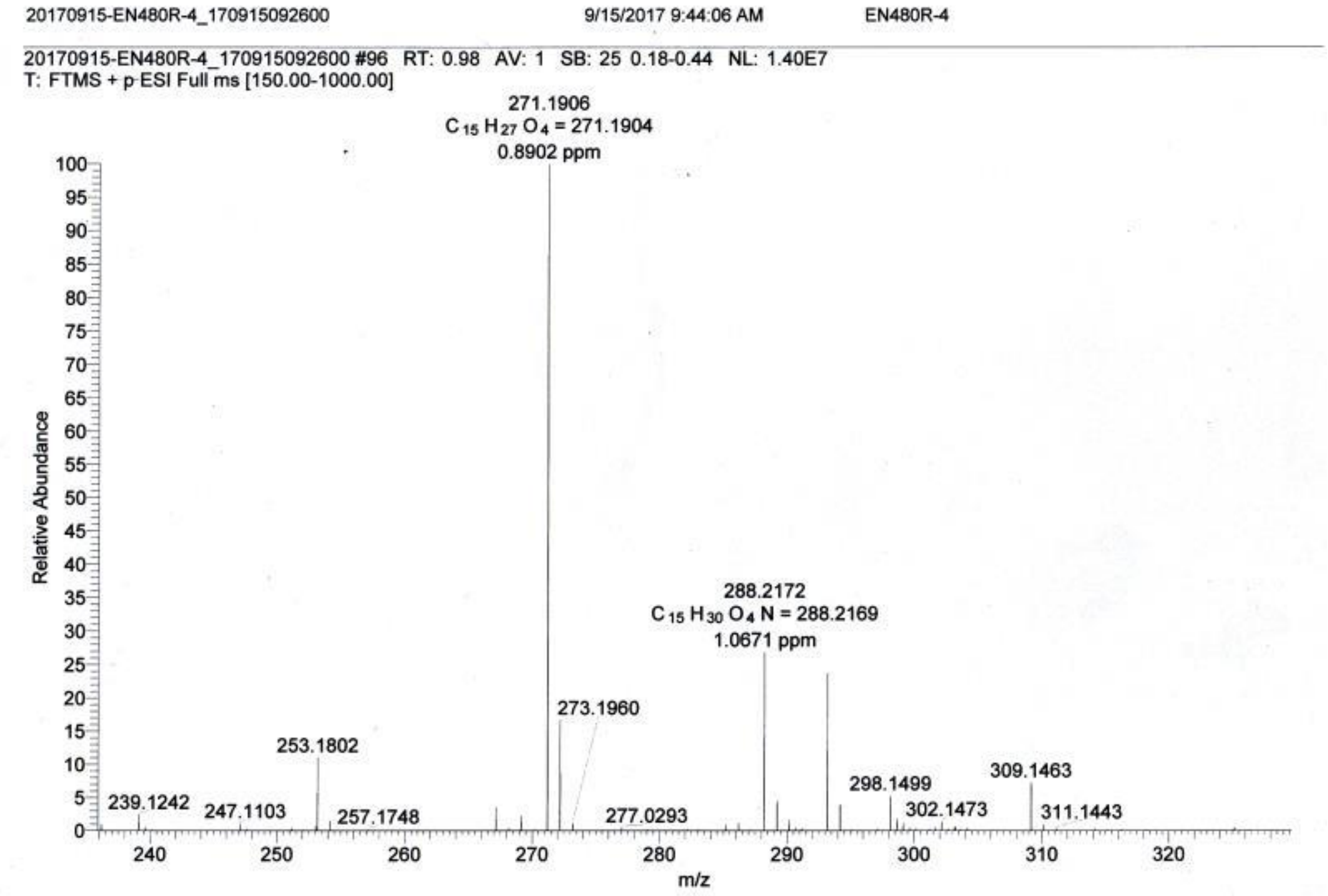

Figure S2. ^{1}H NMR (500 MHz, DMSO-d_6) spectrum of compound **1**.

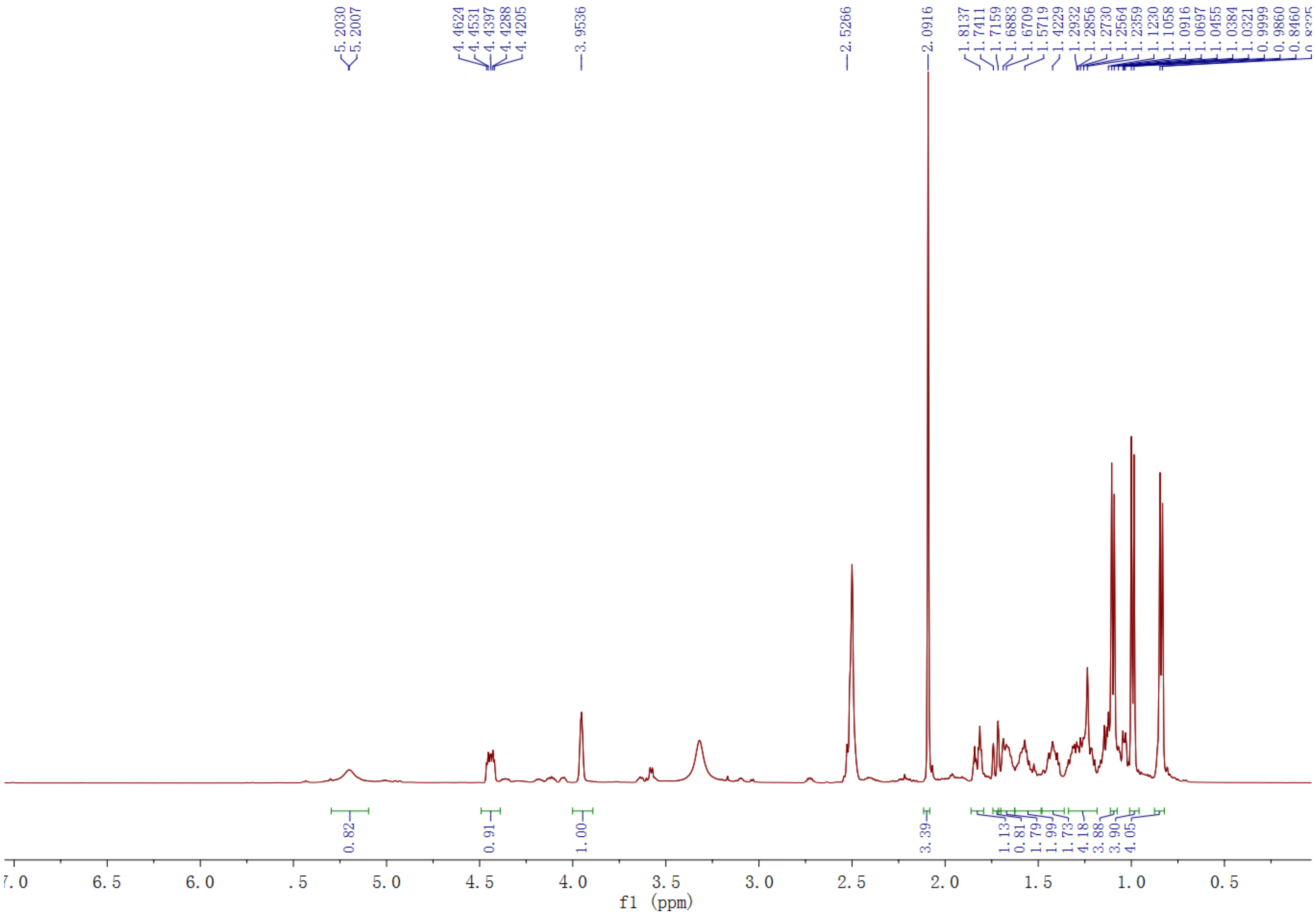

Figure S3. ^{13}C NMR (125 MHz, DMSO-d_6) and DEPT spectra of compound **1**.

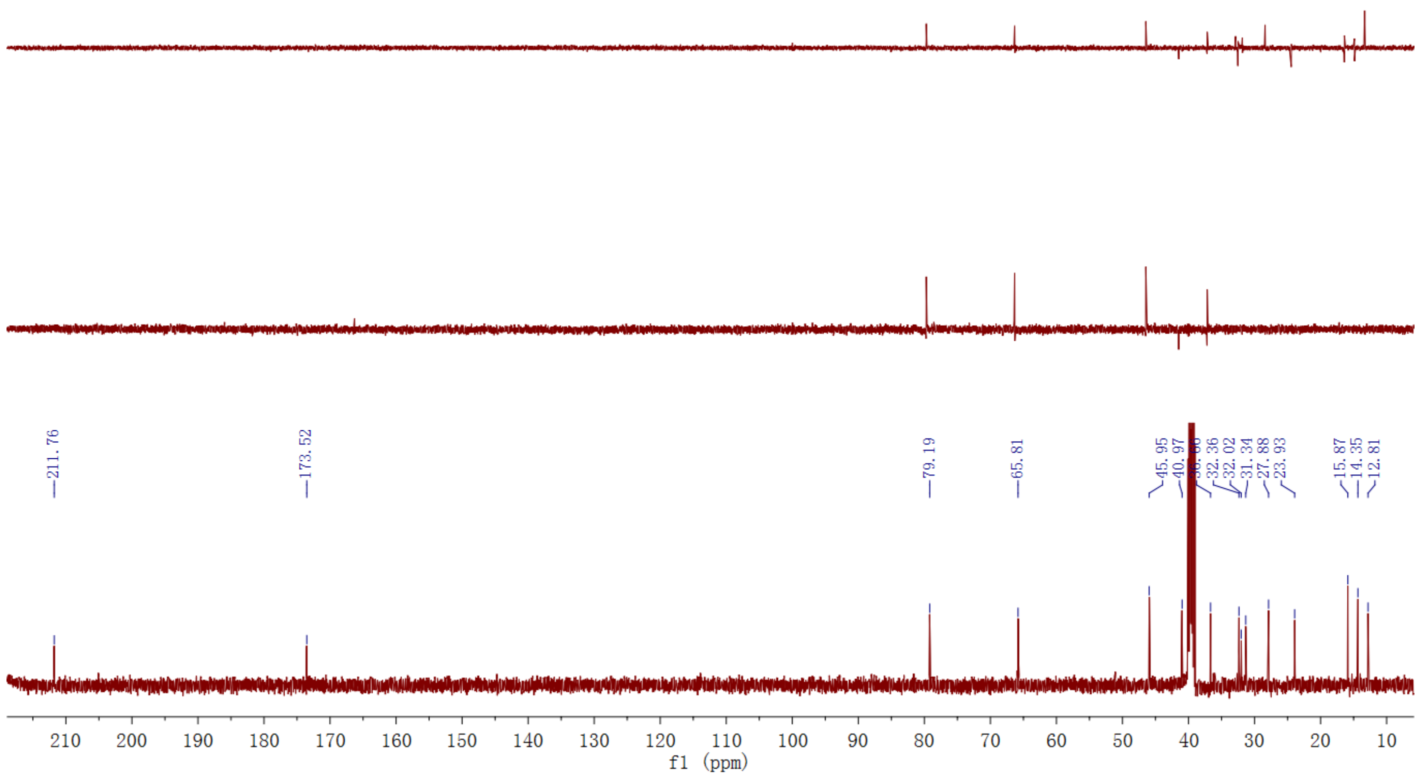

Figure S4. COSY spectrum of compound **1**.

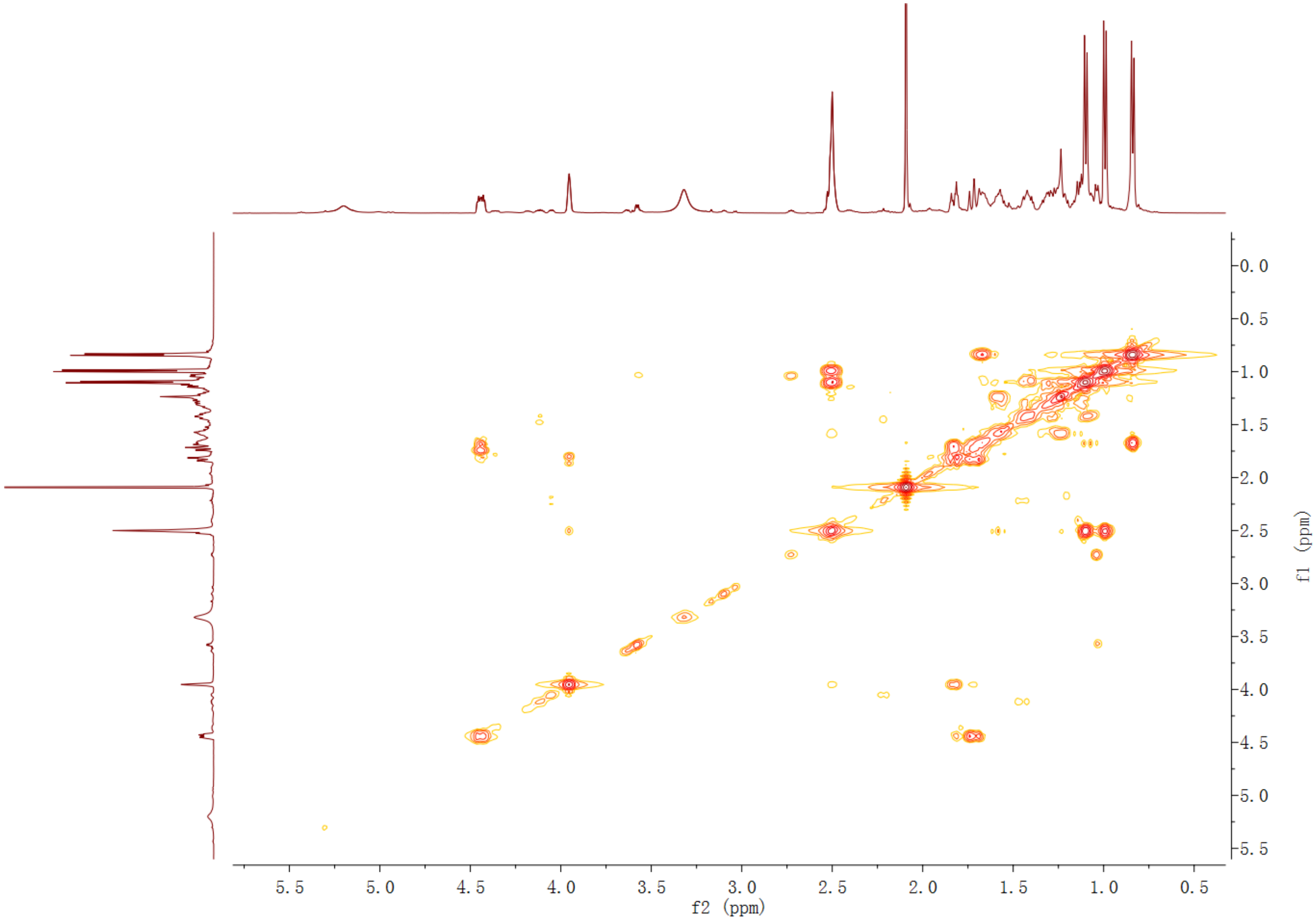

Figure S5. HMBC spectrum of compound **1**.

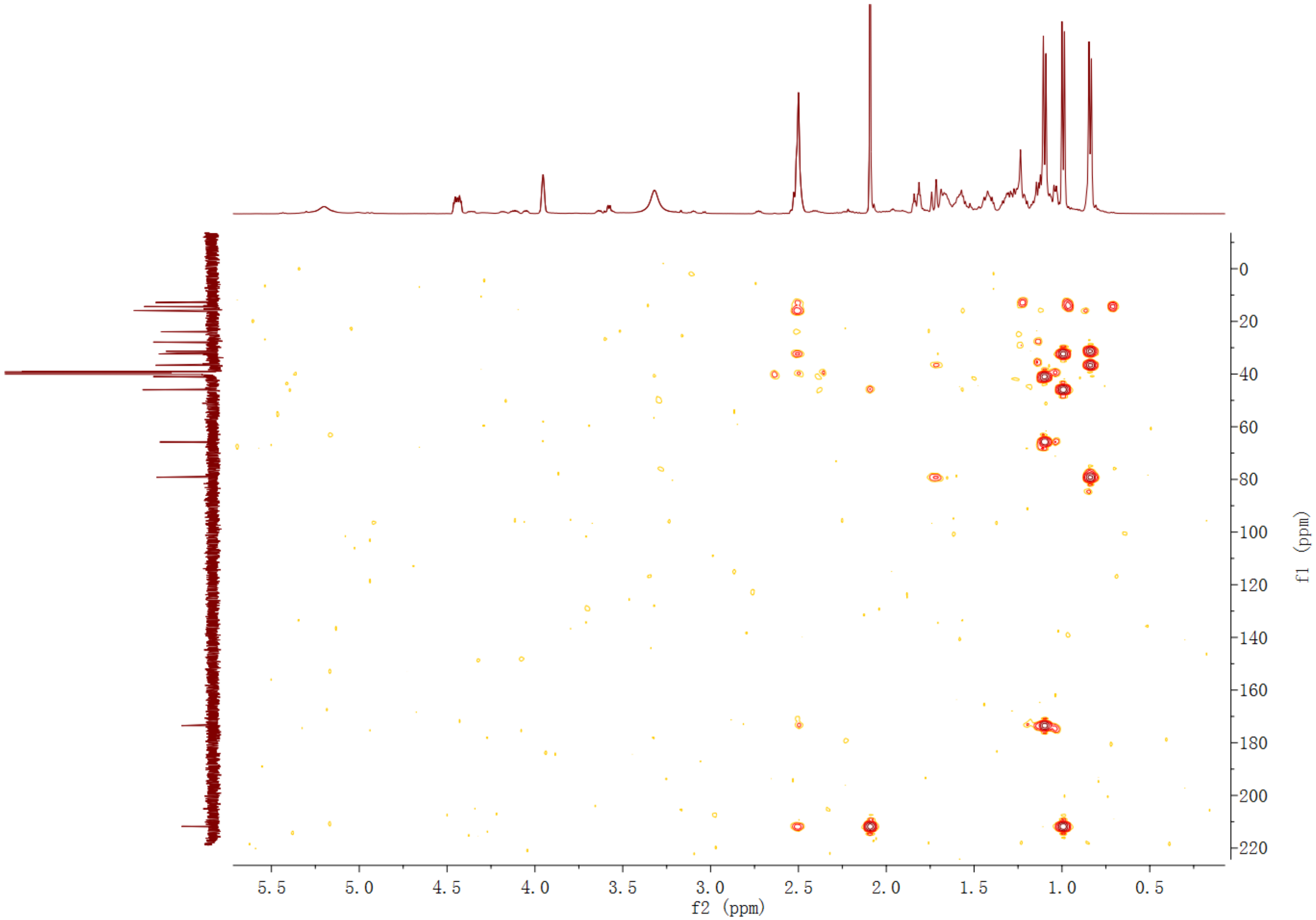

Figure S6. NOESY spectrum of compound **1**.

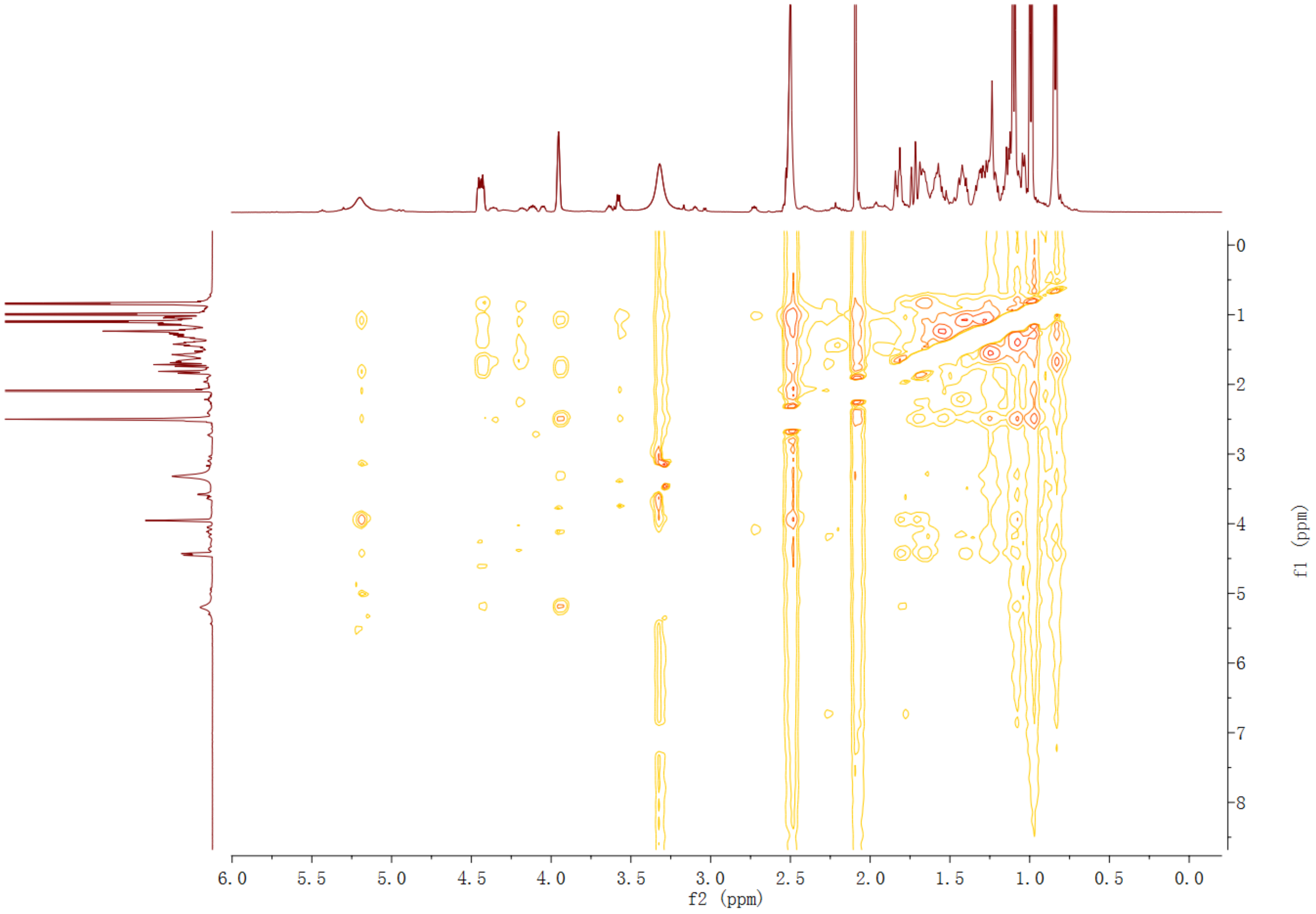

Figure S7. ^{1}H NMR (500 MHz, DMSO-d_6) spectrum of compound **2**.

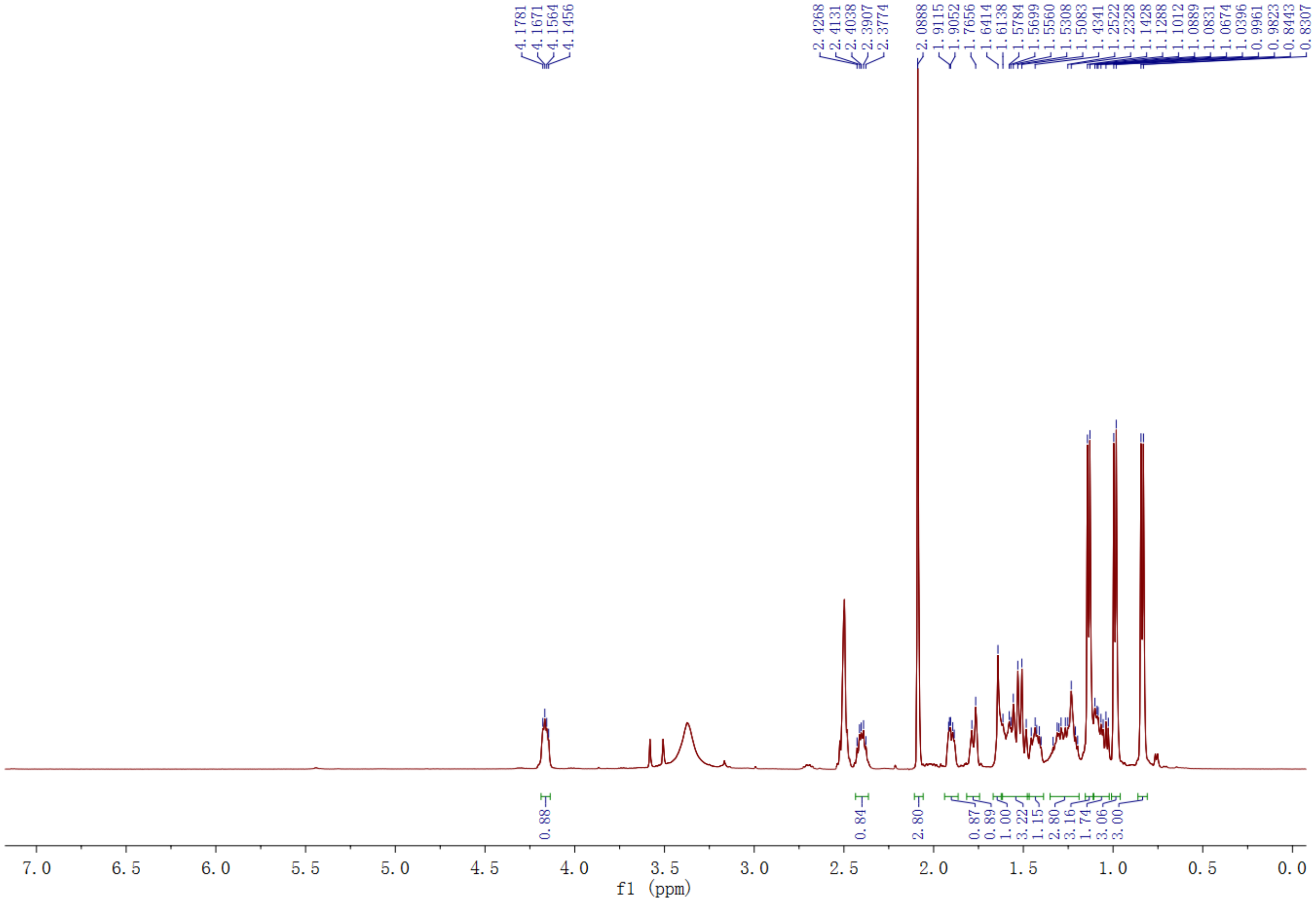

Figure S8. ^{13}C NMR (125 MHz, DMSO-d_6) and DEPT spectra of compound **2**.

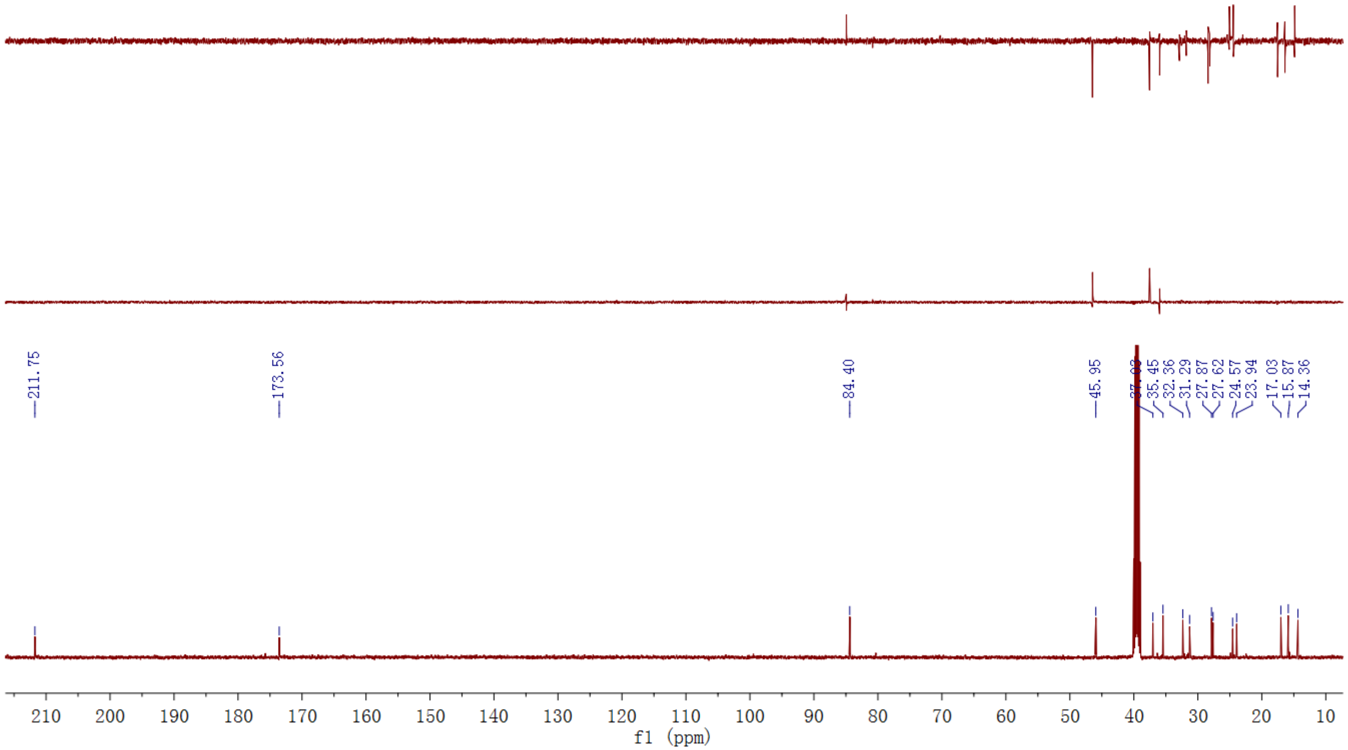

Figure S9. COSY spectrum of compound **2**.

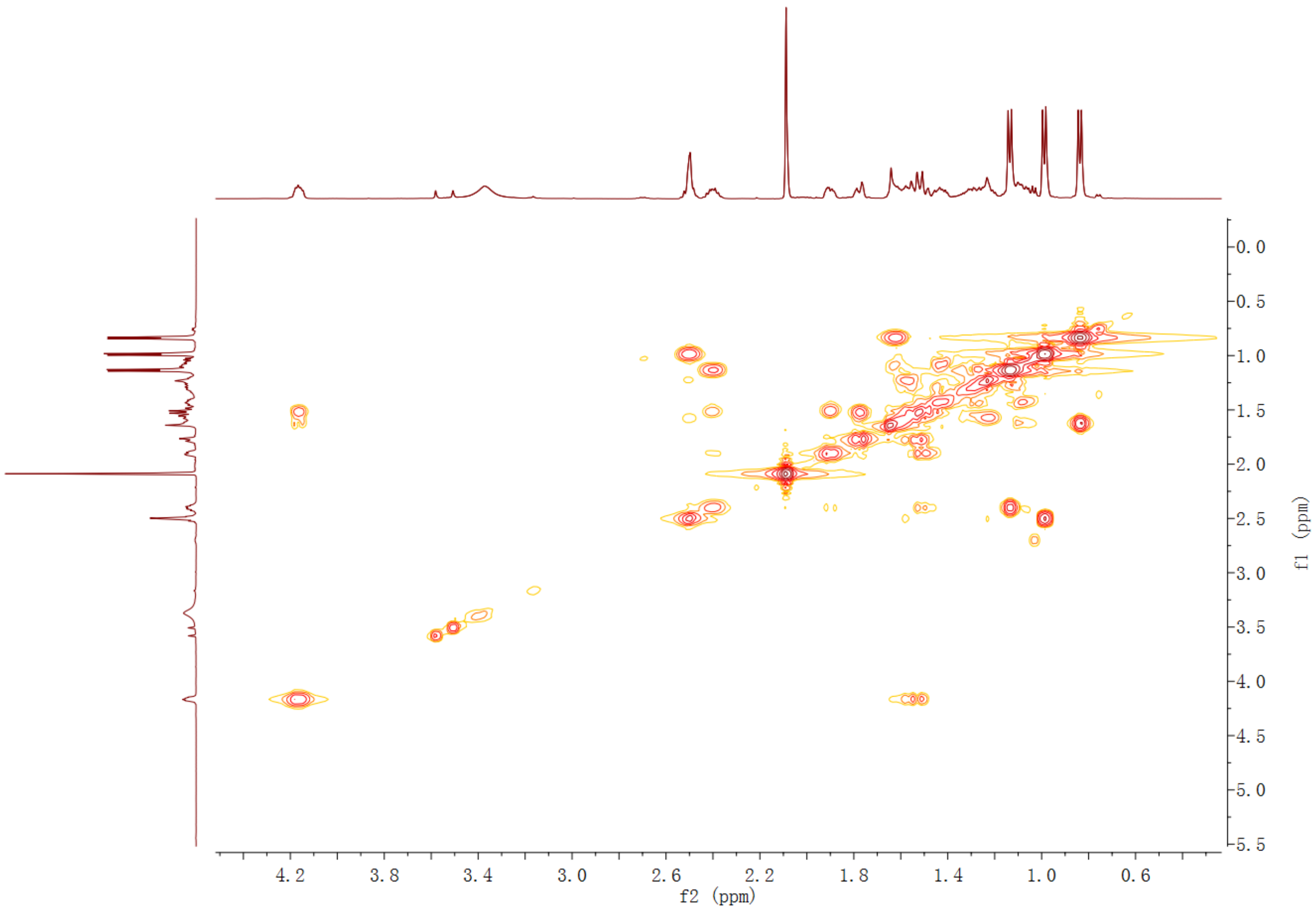

Figure S10. HMBC spectrum of compound **2**.

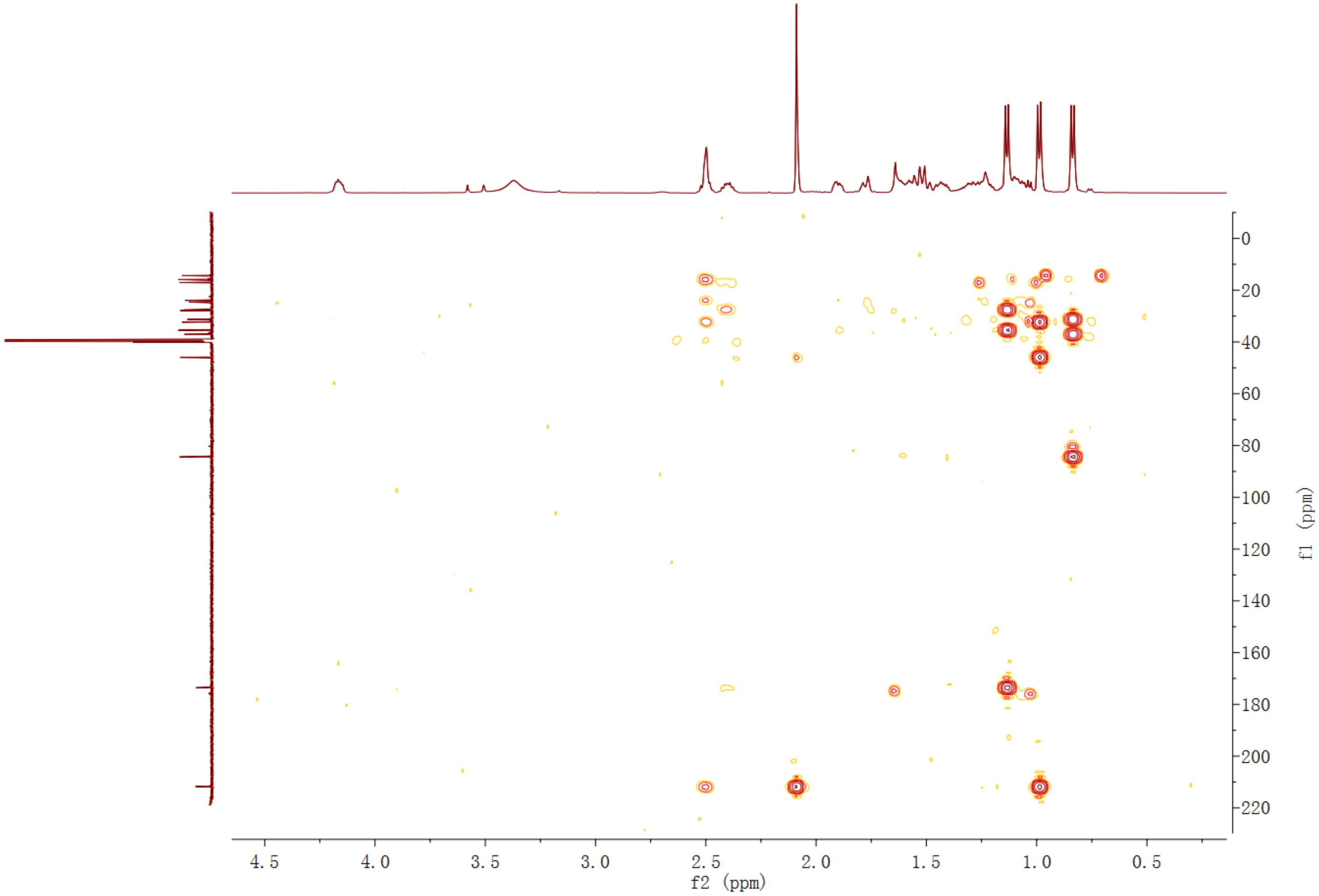

Figure S11. NOESY spectrum of compound **2**.

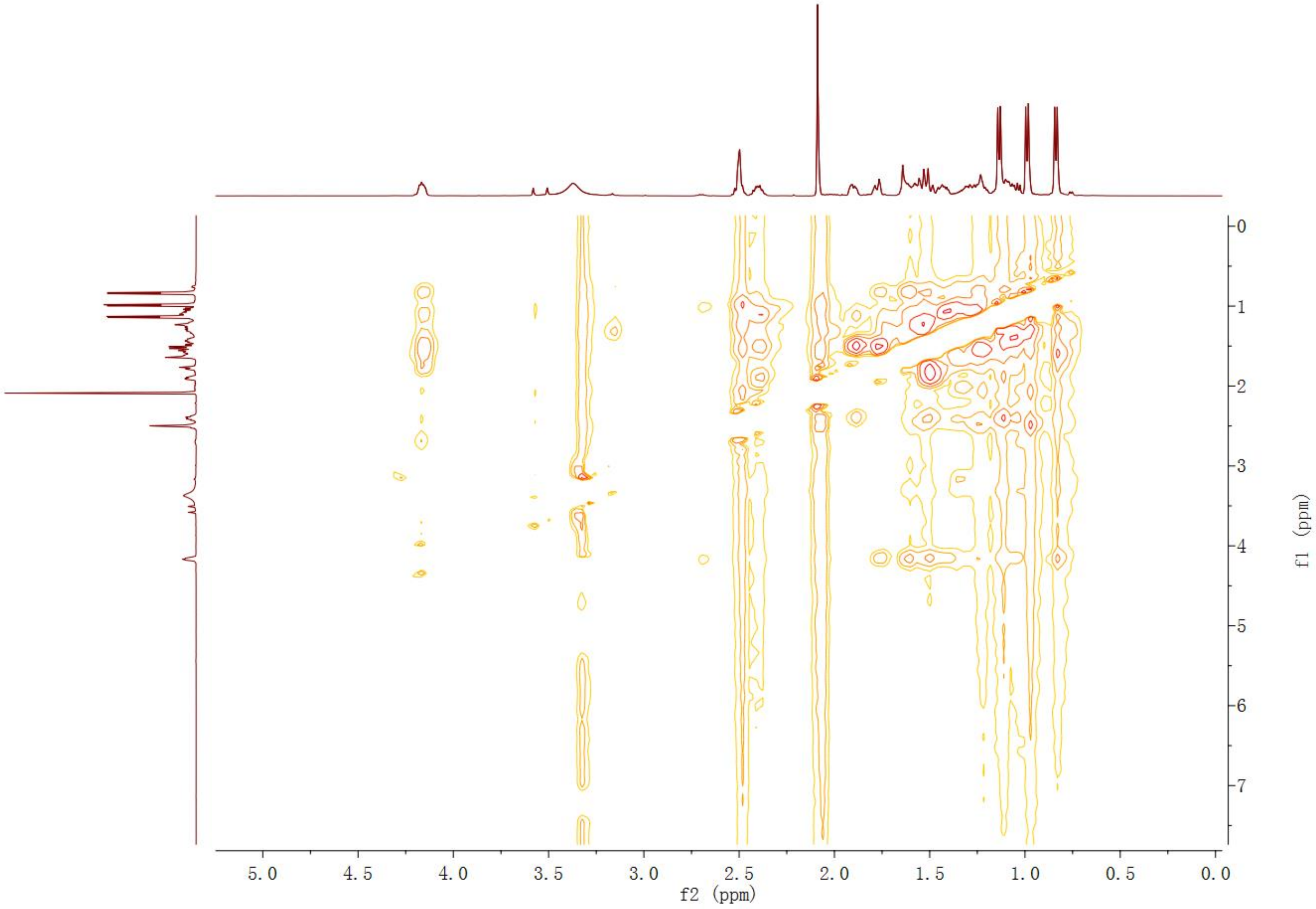

Figure S12. ^{1}H NMR (500 MHz, DMSO-d_6) spectrum of compound **3**.

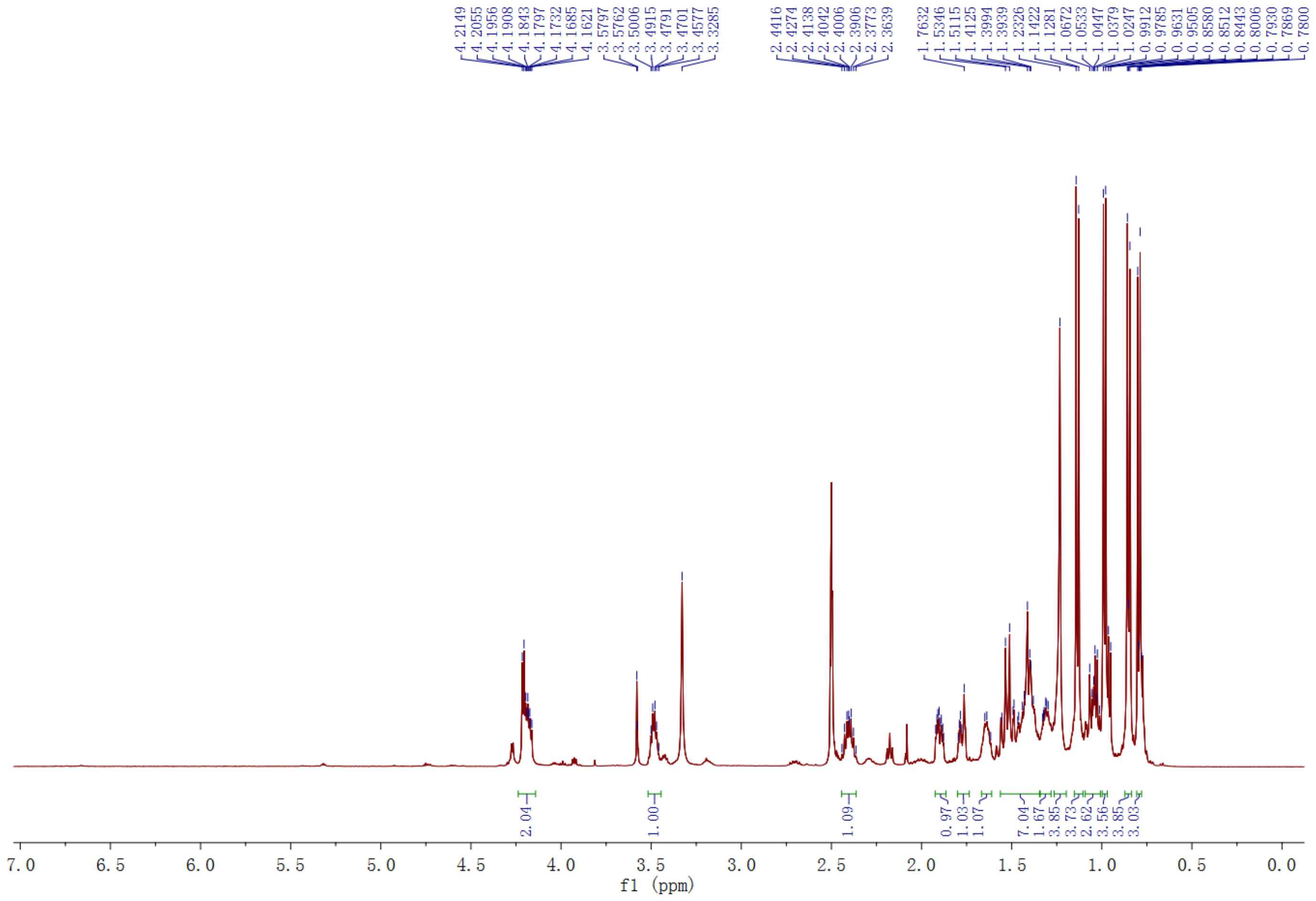

Figure S13. ^{13}C NMR (125 MHz, DMSO-d_6) and DEPT spectra of compound **3**.

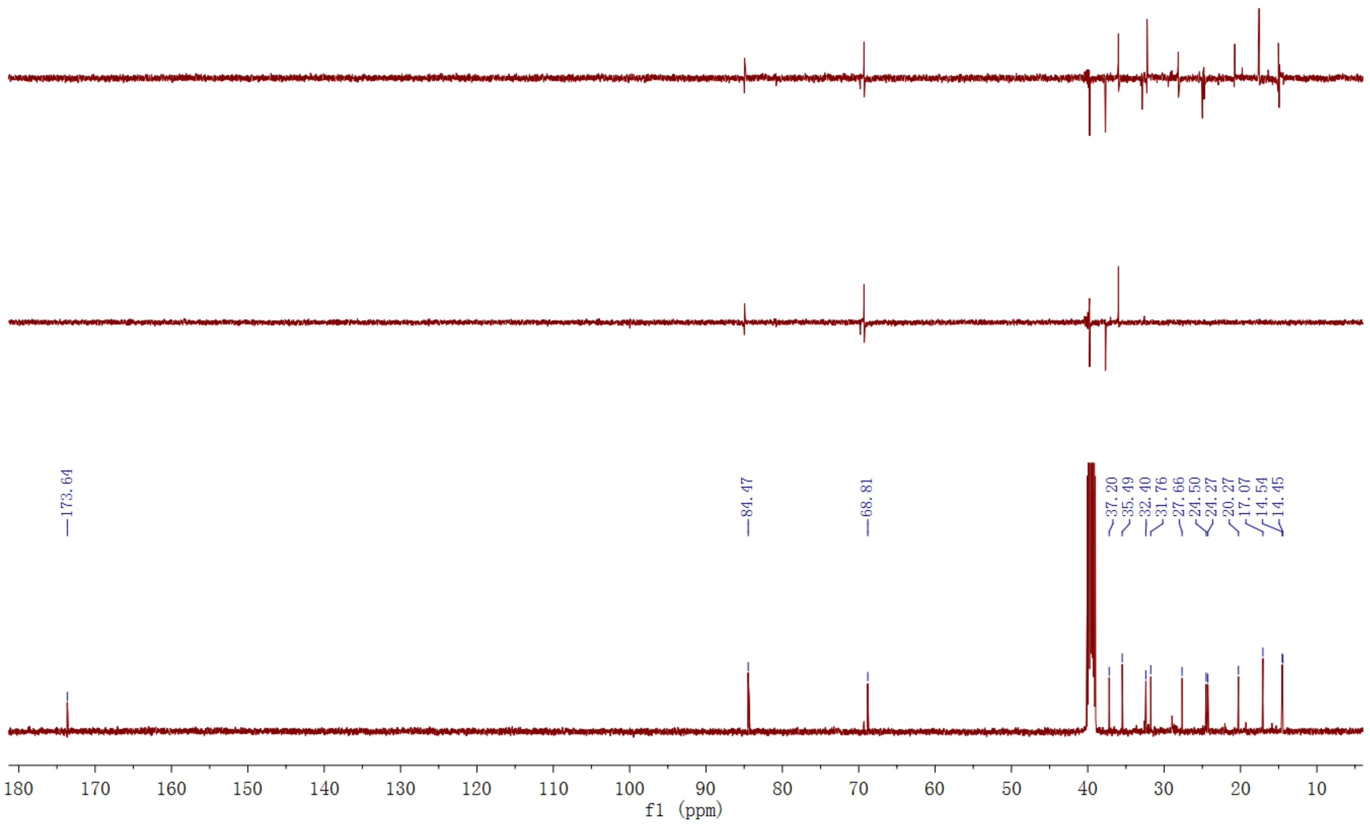

Figure S14. COSY spectrum of compound **3**.

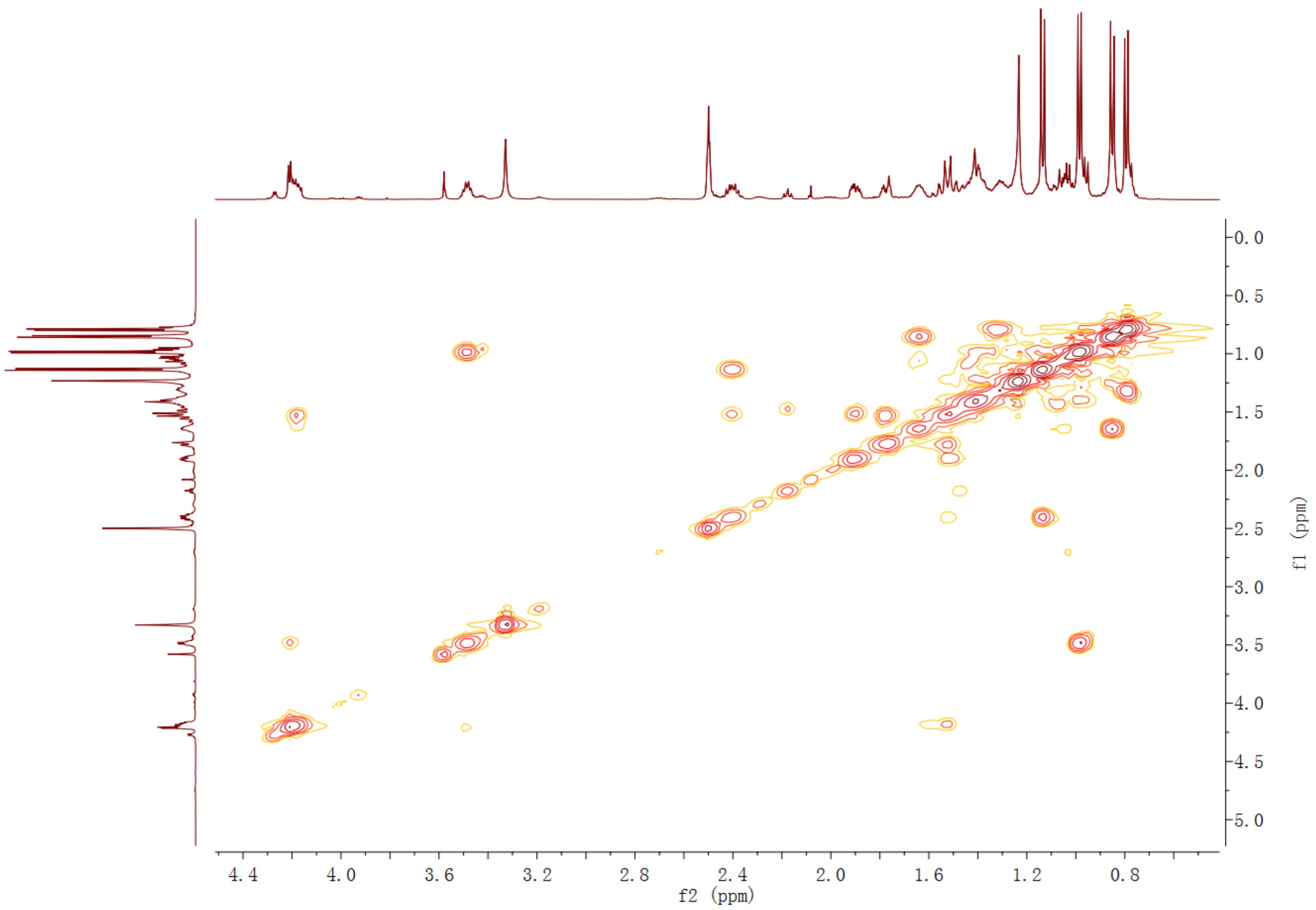

Figure S15. HMBC spectrum of compound **3**.

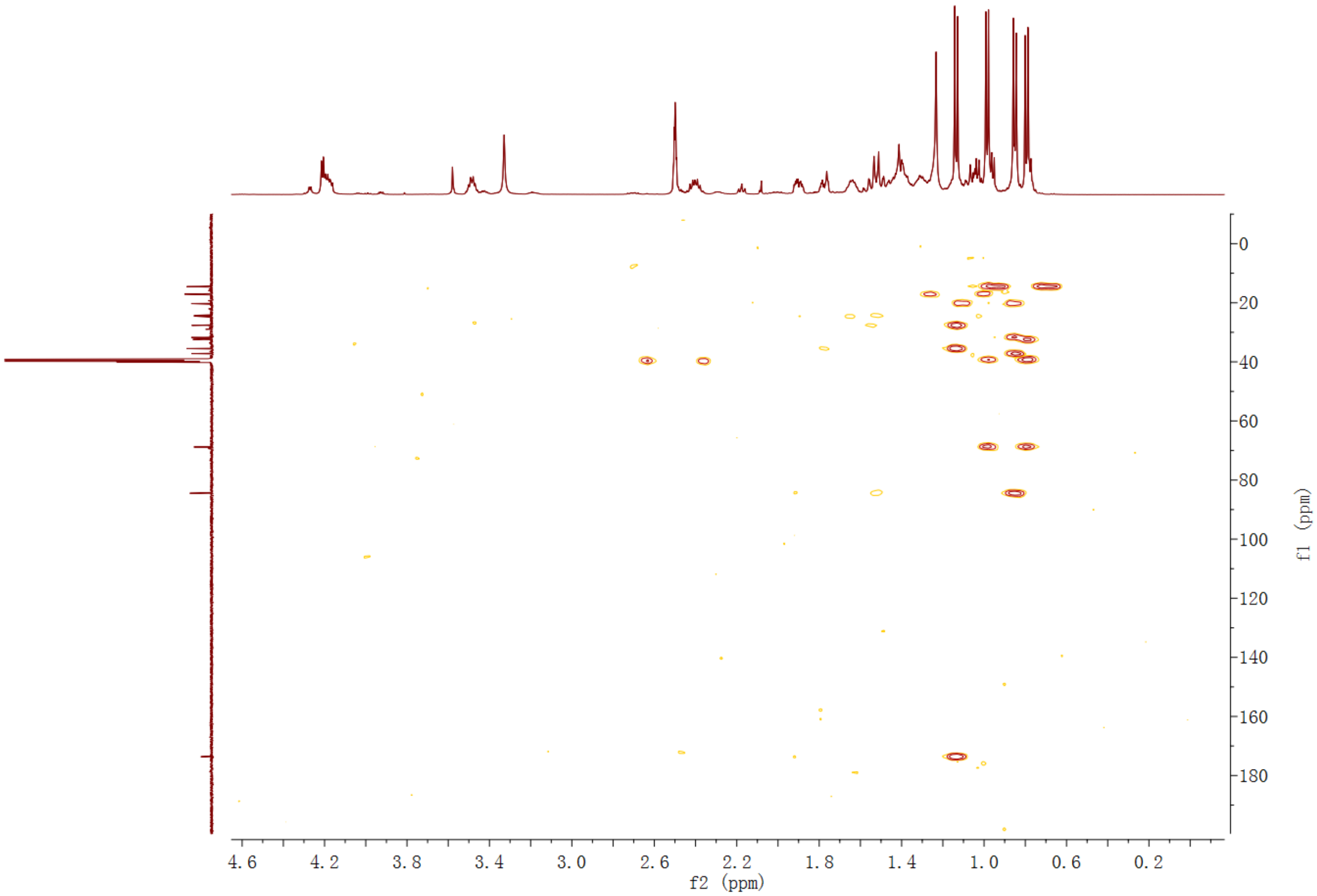

Figure S16. NOESY spectrum of compound **3**.

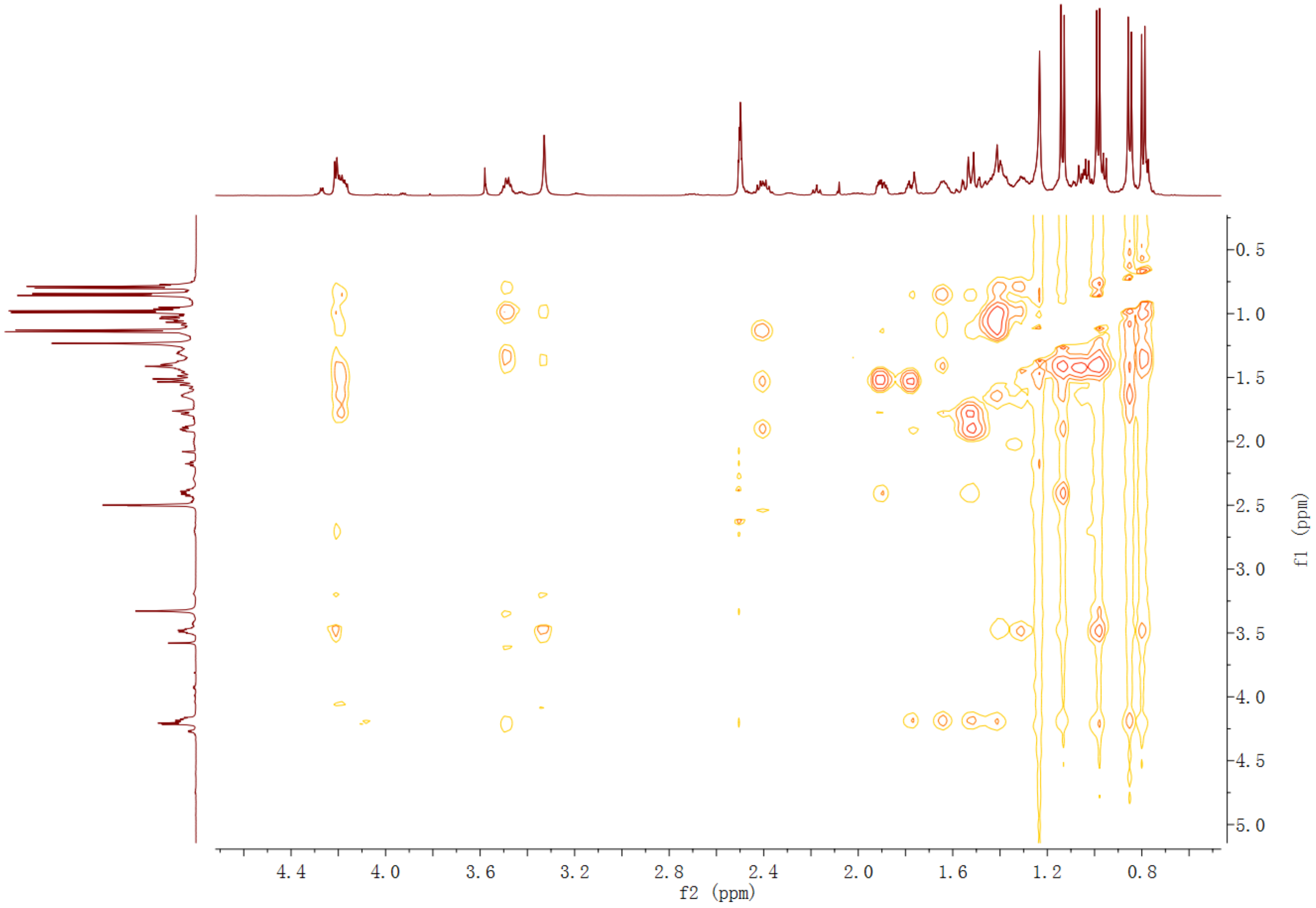

Figure S17. DP4+ probability Excel sheets of compound **1**.

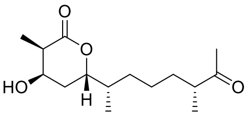

Isomer 1 (**1a**)

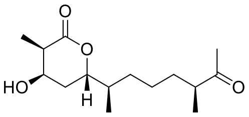

Isomer 2 (**1b**)

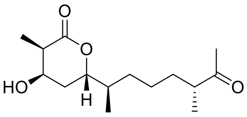

Isomer 3 (**1c**)

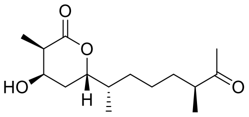

Isomer 4 (**1d**)

	A	B	C	D	E	F	G	H
1	Functional		Solvent?		Basis Set		Type of Data	
2	mPW1PW91		PCM		6-31+G(d,p)		Unscaled Shifts	
3								
12			DP4+	0.00%	0.00%	0.90%	99.10%	-
14	Nuclei	sp2?	xperimenta	Isomer 1	Isomer 2	Isomer 3	Isomer 4	Isomer 5
15	C	x	173.50	153.95127	137.9	149.33536	158.059	
16	C		41.00	32.233057	28.5	31.128696	32.5385	
17	C		65.80	57.033378	51.0	55.46348	58.5199	
18	C		23.90	22.626703	17.6	17.433844	24.3992	
19	C		79.20	64.449366	56.0	60.448952	66.8474	
20	C		36.70	25.560948	22.8	23.386942	26.9897	
21	C		31.30	17.038785	18.4	18.39028	20.2375	
22	C		32.00	7.463994	10.8	11.586476	8.9387	
23	C		32.40	22.291527	18.5	20.897884	21.6212	
24	C		46.00	35.251684	30.3	33.931148	35.601	
25	C	x	211.80	197.36838	175.3	190.34108	201.442	
26	C		27.90	12.338559	15.31	12.590458	17.9286	
27	C		12.80	1.420422	1.08	1.438462	1.3618	
28	C		14.40	2.662186	1.77	1.897212	3.4039	
29	C		15.90	5.43217	6.00	5.417534	7.142	
30								
31	H		2.52	2.617065	2.33	2.5351	2.6573	
32	H		3.95	4.125029	3.66	4.017828	4.2262	
33	H		1.73	2.976471	1.91	2.466662	2.369	
34	H		1.82	1.670472	1.62	1.764934	1.7965	
35	H		4.44	4.253821	3.99	4.492554	4.3815	
36	H		1.68	1.999574	1.95	2.181596	2.0009	
37	H		1.14	1.456267	1.28	1.779472	1.6536	
38	H		1.14	2.08251	1.62	1.434424	1.8054	
39	H		1.26	1.69158	1.26	1.279544	1.5095	
40	H		1.26	1.007941	1.128711	1.466634	1.0731	
41	H		1.14	2.087465	1.804595	1.967878	1.941	
42	H		1.55	1.567643	1.181885	1.745064	1.3687	
43	H		2.52	2.784747	2.771949	2.718346	3.144	
44	H		2.09	2.399196	2.264319	2.3263	2.5478	
45	H		1.10	1.673229	1.469468	1.617338	1.6845	
46	H		0.84	1.354612	1.044125	1.13101	1.4121	
47	H		0.98	4.194075	1.217849	1.366996	1.4019	
48								
49								
50								

Main | Detailed Results

Figure S18. DP4+ probability Excel sheets of compound **2**.

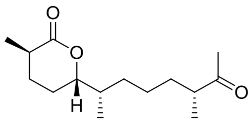

Isomer 1 (**2a**)

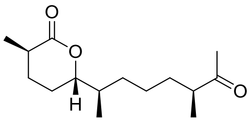

Isomer 2 (**2b**)

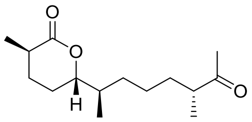

Isomer 3 (**2c**)

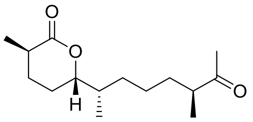

Isomer 4 (**2d**)

	A	B	C	D	E	F	G	H
1	Functional		Solvent?		Basis Set		Type of Data	
2	mPW1PW91		PCM		6-31+G(d,p)		Shielding Tensors	
3								
12			DP4+	100.00%	0.00%	0.00%	0.00%	-
14	Nuclei	sp2?	xperimenta	Isomer 1	Isomer 2	Isomer 3	Isomer 4	Isomer 5
15	C	x	173.56	24.1242	23.011959	23.60158	22.691946	
16	C		35.45	156.298	155.23803	154.65154	154.88628	
17	C		27.87	165.233	163.47447	163.57321	162.89739	
18	C		27.62	172.212	164.67126	165.96687	157.15527	
19	C		84.40	108.86	111.07848	109.37328	114.57903	
20	C		37.03	155.547	153.6294	155.92468	157.61283	
21	C		31.29	160.452	158.559762	160.73202	160.84836	
22	C		24.57	166.418	173.01048	168.78019	167.93736	
23	C		32.36	159.953	160.218	159.10895	162.05187	
24	C		45.95	146.784	148.26333	145.04588	145.89546	
25	C	x	211.75	-20.1235	-19.693377	-19.56505	-19.370673	
26	C		23.94	163.818	163.3017	162.34056	162.59898	
27	C		17.03	177.838	176.19966	175.88769	175.96035	
28	C		14.36	176.786	178.31226	172.74894	178.87335	
29	C		15.87	175.901	173.33928	173.89608	173.58252	
30								
31	H		2.4	29.2261	28.845987	28.882677	28.82664	
32	H		1.9	30.0046	29.683317	29.67089	29.488662	
33	H		1.56	29.6206	29.35143	29.313048	29.269023	
34	H		1.78	29.8232	29.347659	29.621675	29.631207	
35	H		1.57	29.9074	29.756562	29.449015	29.267028	
36	H		4.17	27.1408	27.080199	26.775889	26.573841	
37	H		1.64	29.5896	29.469129	29.416547	29.428302	
38	H		1.08	30.3558	29.060325	30.011322	29.643042	
39	H		1.08	30.169	30.264225	29.568942	30.025413	
40	H		1.27	30.5775	30.229719	29.999067	30.031506	
41	H		1.27	29.8764	29.981016	30.304288	30.046134	
42	H		1.23	30.5435	29.237664	30.270589	29.341563	
43	H		1.55	30.4289	30.302715	29.542875	30.294108	
44	H		2.5	28.79	28.143639	28.477487	28.455996	
45	H		2.09	29.383	28.786152	29.069633	29.033268	
46	H		1.14	30.374	30.072564	30.049355	30.021528	
47	H		0.84	30.6448	30.367866	30.182853	30.367578	
48	H		0.99	30.5123	30.202833	30.222871	30.239934	
49								
50								

Main | Detailed Results

Figure S19. DP4+ probability Excel sheets of compound **3.**

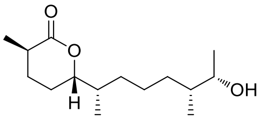

Isomer 1 (**3a**)

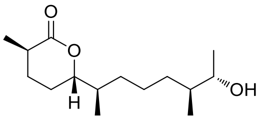

Isomer 2 (**3b**)

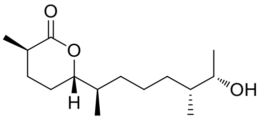

Isomer 3 (**3c**)

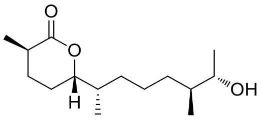

Isomer 4 (**3d**)

	A	B	C	D	E	F	G	H
1	Functional		Solvent?		Basis Set		Type of Data	
2	mPW1PW91		PCM		6-31+G(d,p)		Unscaled Shifts	
3								
12			DP4+	0.00%	0.00%	100.00%	0.00%	-
14	Nuclei	sp2?	xperimenta	Isomer 1	Isomer 2	Isomer 3	Isomer 4	Isomer 5
15	C	x	173.6	21.58	19.9	22.47462564	19.444353	
16	C		35.5	156.545	120.3	156.3809744	125.45432	
17	C		27.7	165.809	129.9	165.752859	134.28391	
18	C		24.5	172.322	165.9	169.9982179	164.67292	
19	C		84.5	107.328	111.0	110.5095385	110.96522	
20	C		37.2	160.138	155.2	155.933641	1064.59344	
21	C		31.8	162.037	165.0	160.6638846	161.65297	
22	C		24.3	174.669	175.3	171.8792949	172.16078	
23	C		32.4	164.561	163.8	161.6007308	161.29671	
24	C		40	153.406	157.9	153.489141	156.18003	
25	C		68.8	127.373	121.4	122.9007821	119.85295	
26	C		20.3	172.519	179.88	173.0329615	178.08411	
27	C		17.1	177.447	178.52	178.0852051	176.79942	
28	C		14.5	175.539	179.82	179.9491026	178.08615	
29	C		14.5	178.984	175.62	178.303141	175.39964	
30								
31	H		2.4	29.0578	29.07	29.08405897	28.773343	
32	H		1.9	29.9963	29.87	29.96154103	29.56458	
33	H		1.53	29.5846	29.60	29.63113718	29.272391	
34	H		1.78	29.8045	29.55	29.73214615	29.431241	
35	H		1.51	29.8008	29.94	29.96466538	29.494816	
36	H		4.17	27.0092	27.29	27.1089359	26.925817	
37	H		1.638	29.1799	29.71	29.75250385	29.487766	
38	H		1.05	30.3459	29.27	30.03429359	29.103964	
39	H		1.05	29.7921	29.50	30.41345897	29.388	
40	H		1.4	30.3536	30.17996	29.91086154	28.866932	
41	H		1.4	30.1884	30.24424	30.33316538	30.026083	
42	H		1.03	28.8998	29.94076	30.10935641	29.497017	
43	H		0.96	30.6552	30.48782	29.98940256	30.320824	
44	H		1.31	30.1045	29.44944	30.09032692	29.159755	
45	H		3.49	28.0405	27.58348	28.14976538	27.280718	
46	H		0.97	30.523	30.5468	30.38819872	30.209437	
47	H		1.14	30.3169	30.40494	30.26057051	30.099258	
48	H		0.85	30.6811	30.60436	30.70603077	30.306998	
49	H		0.79	30.7608	30.8007	30.76496282	30.504367	
50								

Main | Detailed Results
